# P-1666. Hematology-Oncology Unit-Specific Antibiogram: A Decade of Experience at a Large Academic Medical Center

**DOI:** 10.1093/ofid/ofae631.1832

**Published:** 2025-01-29

**Authors:** Josh Clement, Christina G Rivera, Nicolynn Cole, Audrey N Schuetz, Lynn L Estes

**Affiliations:** The Mount Sinai Hospital, New York, New York; Mayo Clinic, Rochester, Minnesota; Mayo Clinic, Rochester, Minnesota; Mayo Clinic, Rochester, Minnesota; Mayo Clinic - Rochester, Rochester, Minnesota

## Abstract

**Background:**

Antibiograms are an important stewardship tool that can inform optimal empiric therapy; most antibiograms are hospital-wide. Unit-specific antibiogram data for unique patient populations including intensive care unit and pediatrics is robust; however, published experience with unit-specific antibiograms in the hematology-oncology (heme-onc) population is limited. These patients are unique because of the common use of antimicrobial prophylaxis, prolonged broad-spectrum antibiotics, and an immunocompromised state. We sought to examine the trends in antibiogram antimicrobial susceptibility over time.

**Figure:**
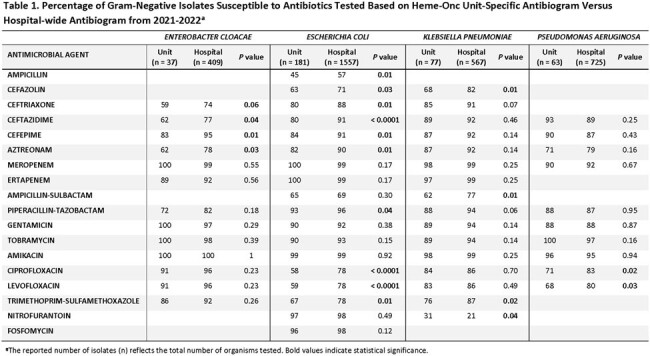

**Methods:**

This retrospective quality initiative included microbiology results (first isolates per patient per timeframe) from Mayo Clinic Rochester from 2012 to 2022. Eligible cultures were those identified as being obtained from one of 8 hematology, oncology, or bone marrow transplant inpatient units. Agar dilution susceptibility testing and Clinical and Laboratory Standards Institute (CLSI) breakpoints were used. Screening PCRs (eg, VRE PCR) were excluded. Culture and susceptibilities were identified by an EMR based report, compiled annually, and reported for two-year periods. Susceptibility rates were compared using the Chi-square test and Chi-square analysis for trends.

**Figure:**
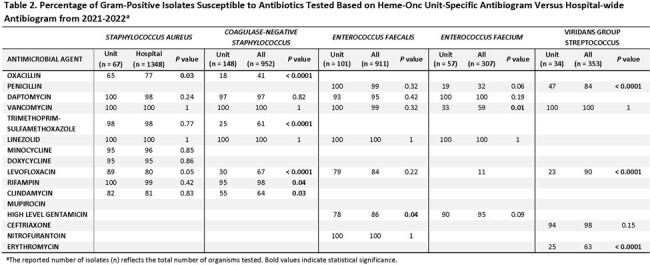

**Results:**

There were 4 gram-negative and 5 gram-positive organism groups that met CLSI antibiogram criteria of ≥ 30 isolates and were included (tables 1-4). There were several significant differences in comparison to the hospital-wide antibiogram, with lower antibiotic susceptibility rates in the heme-onc units for multiple organisms, notably fluoroquinolones to *E.coli* and *P. aeruginosa* and several cephalosporins to Enterobacterales as well as penicillin and levofloxacin to Viridans group Streptococcus and vancomycin to *E. faecium* (tables 1&2). Over the period of 2012-2013 to 2021-2022, susceptibility rates remained stable among the heme-onc antibiogram, with few significant changes (tables 3&4).

**Figure:**
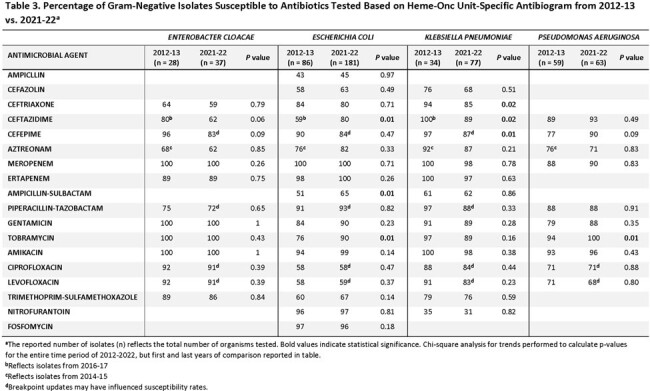

**Conclusion:**

Heme-onc antibiograms illustrated susceptibility rate differences compared to our hospital-wide antibiograms, particularly for antimicrobials used as prophylaxis. Through population-specific antibiograms, providers may be able to improve empiric antibiotic selection.

**Figure:**
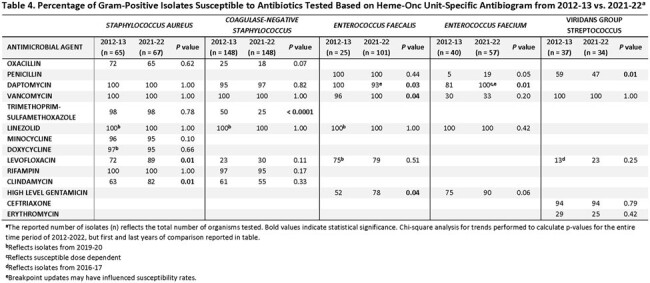

**Disclosures:**

**Christina G. Rivera (O'Connor), Pharm.D**, Gilead Sciences: Board Member

